# Understanding the epidemiology, clinical characteristics, knowledge and barriers to treatment and prevention of malaria among returning international laborers in northern Vietnam: a mixed-methods study

**DOI:** 10.1186/s12879-022-07322-5

**Published:** 2022-05-13

**Authors:** Kimberly A. Edgel, Sara Canavati, Hoi Thi Le, Tho Huy Tran, Kinh Van Nguyen, Trung Vu Nguyen, Nga Thi Nguyen, Hoa Mai Tran, Thang Duc Ngo, Duong Thanh Tran, Binh Thi Huong Nguyen, Long Khanh Tran, Thu Minh Nguyen, Rosalie J. Whedbee, Ekaterina I. Milgotina, Nicholas J. Martin

**Affiliations:** 1 U.S. Naval Medical Research Unit TWO, Singapore, Singapore; 2 Vysnova Partners, Inc., Bethesda, MD USA; 3 Centre for Biomedical Research, Burnet Institute, Melbourne, Australia; 4 National Hospital for Tropical Diseases, Hanoi, Vietnam; 5 Parasitology and Entomology (NIMPE), National Institute of Malariology, Hanoi, Vietnam; 6 Hanoi Medical University, Hanoi, Vietnam; 7 Global Scientific Solutions for Health, Baltimore, MD USA

**Keywords:** Vietnam, *Plasmodium falciparum*, *Plasmodium vivax*, *Plasmodium ovale*, *Plasmodium malariae*, Migration, Population movement, Key populations, Malaria importation, Malaria elimination

## Abstract

**Background:**

With the decline in local malaria transmission in Vietnam as a result of the National Malaria Control Program (NMCP) elimination activities, a greater focus on the importation and potential reintroduction of transmission are essential to support malaria elimination objectives.

**Methods:**

We conducted a multi-method assessment of the demographics, epidemiology, and clinical characteristics of imported malaria among international laborers returning from African or Southeast Asian countries to Vietnam. Firstly, we conducted a retrospective review of hospital records of patients from January 2014 to December 2016. Secondly, we conducted a mixed-methods prospective study for malaria patients admitted to the study sites from January 2017 to May 2018 using a structured survey with blood sample collection for PCR analysis and in-depth interviews. Data triangulation of the qualitative and quantitative data was used during analysis.

**Results:**

International laborers were young (median age 33.0 years IQR 28.0–39.5 years), predominantly male (92%) adults returning mostly from the African continent (84%) who stayed abroad for prolonged periods (median time 13.5 months; IQR 6.0–331.5 months) and were involved in occupations that exposed them to a higher risk of malaria infection. Epidemiological trends were also similar amongst study strands and included the importation of *Plasmodium falciparum* primarily from African countries and *P. vivax* from Southeast Asian countries. Of 11 *P. malariae* and *P. ovale* infections across two study strands, 10 were imported from the African continent. Participants in the qualitative arm demonstrated limited knowledge about malaria prior to travelling abroad, but reported knowledge transformation through personal or co-worker’s experience while abroad. Interestingly, those who had a greater understanding of the severity of malaria presented to the hospital for treatment sooner than those who did not; median of 3 days (IQR 2.0–7.0 days) versus 5 days (IQR 4.0–9.5 days) respectively.

**Conclusion:**

To address the challenges to malaria elimination raised by a growing Vietnamese international labor force, consideration should be given to appropriately targeted interventions and malaria prevention strategies that cover key stages of migration including pre-departure education and awareness, in-country prevention and prophylaxis, and malaria screening upon return.

**Supplementary Information:**

The online version contains supplementary material available at 10.1186/s12879-022-07322-5.

## Background

The Vietnam National Malaria Control Program (NMCP) is focused on malaria elimination [1–3] which resulted in a 77% decline in malaria cases between 2012 and 2017 and has surpassed its strategic targets for 2020 [1]. However, reported cases mostly flatlined in 2017 to 2018, with reports indicating increased cases in 2018 [1]. As local malaria transmission declines in response to elimination activities, a greater focus is needed on the possibility for the reintroduction of malaria transmission due to imported cases [4–7]. Imported infections often represent the majority of new cases and can cause resurgences in areas that previously reported no cases, making it critical to effectively address imported cases to achieve elimination [6, 8–10].

In support of Vietnam’s malaria elimination goals, there is currently a focus on the role and threat of imported malaria from Vietnamese citizens pursuing and returning from international labor, particularly in Africa and other Southeast Asian countries. International departure for labor purposes is the most common form of migration for Vietnamese, including those who work abroad for fixed contracts before returning home [11].

Current malaria elimination efforts in Vietnam and Southeast Asia focus on the most prevalent malaria species in the region, *P. falciparum* and *P. vivax *[12, 13], whereas cases imported from other parts of the world have the potential to fall outside of this paradigm. *P. ovale* and *P. malariae* are primarily distributed in sub-Saharan Africa [14, 15] and their appearance in the Greater Mekong Subregion may further complicate elimination efforts [16].

Currently, little understanding exists on the epidemiology of malaria imported into Vietnam from returning international laborers. Recommendations for the NMCP include the need to initiate reporting on the number of imported malaria cases and their country of origin [2]. To improve targeted intervention, a robust understanding of the populations importing infections, including their migration patterns, is required.

## Methods

**Fig. 1:**
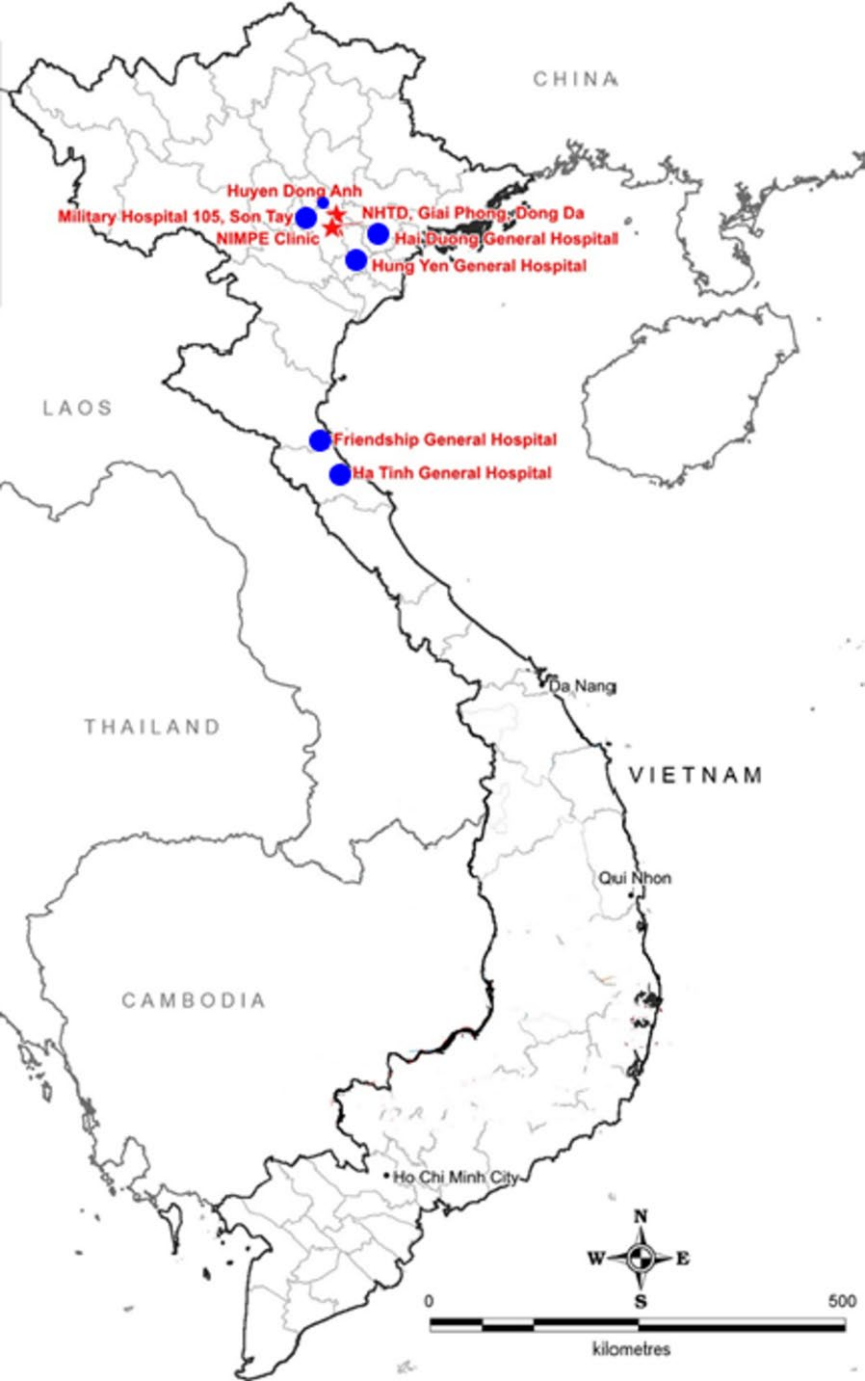
Study sites; (*retrospective study sites denoted by stars while all sites were included in prospective study)*. This map was developed for the purpose of this article

To understand the epidemiology of imported malaria cases in Vietnam among returning international laborers, as well as their health seeking behaviors and travel patterns, a mixed methods assessment was conducted in eight Northern Vietnam hospitals (Fig. 1) from January 2017 to May 2018.

The study was a collaborative effort between the National Hospital of Tropical Diseases (NHTD) and national and provincial level hospitals, including: (1) The National Institute of Malariology, Parasitology and Entomology, (2) Military Hospital 105, (3) General Hospital—Hung Yen Province, (4) General Hospital—Hai Duong Province, (5) Friendship General Hospital—Nghe An Province, (6) General Hospital—Ha Tinh Province. NHTD is a tertiary level teaching hospital, receiving infection related referrals from northern Vietnam and has close ties to the Ministry of Health (MoH). NHTD provided a team of doctors and scientists. In addition, laboratories of NHTD were equipped with appropriate and adequate machines and equipment to serve this study. The NHTD Laboratory Department received the ISO 15189 certification in 2013. Other collaborating hospitals contributed with patient recruitment and sample collection for the study.

The objectives of this study were to: (1) retrospectively describe the epidemiological and clinical characteristics of imported malaria from 2014 to 2016 from all countries and identify risk factors associated with delayed parasite clearance; (2) prospectively describe the clinical and epidemiologic characteristics of confirmed malaria patients returning from African or Southeast Asian countries; and (3) gain an in-depth understanding of patterns of migration, patient treatment-seeking behavior, and knowledge of malaria among returning international laborers throughout the different stages of migration.

### Quantitative retrospective study

#### Data collection

A retrospective review of hospital records for the period between January 2014 and December 2016 at the NHTD and the Vietnam National Institute of Malariology, Parasitology and Entomology (NIMPE) Clinic in Hanoi identified malaria cases in patients who recently returned from African or Southeast Asian countries. The review was conducted to collect and analyze demographic, clinical and epidemiologic characteristics of international travelers with the goal of informing the design of the prospective study and the development of the interview guide for the in-depth, qualitative evaluation of patterns in migration, attitude toward malaria prevention and malaria treatment-seeking behavior, and throughout the different stages of migration: pre-departure, upon arrival and on-return (Additional file 1: In-depth interview guideline).

There was no sample size estimation for this retrospective study. All documented laboratory-confirmed or clinical malaria cases with a recent history of traveling abroad and admission to participating hospitals between January 2014 and December 2016 were included in the study. Demographic, clinical and epidemiological information were extracted from hospital records. A case investigation form was used to collect demographic information, the departure date from Vietnam, countries visited, arrival date to Vietnam, original residence in Vietnam, date of admission, microscopy and malaria rapid diagnostic test (RDT) results, date of parasite clearance after treatment with anti-malarial medicines, fever status after treatment, medications used for malaria treatment, duration of the treatment course, and history of contracting overseas malaria. Data were verified to ensure internal consistency. Efforts were made to find any incomplete records or inconsistencies in reporting. For example, records were screened for duplicates (same patient) and recurrent episodes of malaria cases (return within 28 days post-treatment); no instances of either were identified in the reviewed records.

### Quantitative prospective study

#### Data collection and sampling

A prospective study was conducted at the NHTD and NIMPE in Hanoi from January 2017 to May 2018 to describe the clinical and epidemiologic characteristics of confirmed malaria patients recently returning from African or Southeast Asian countries using case report form (Additional file 2: Case report form).

All patients admitted to the study sites who (1) exhibited at least three clinical symptoms of malaria including chills, fever, and sweats; (2) met the criteria for treatment outlined in the Vietnamese National Malaria Treatment Guideliness (Decision 4845/QD-BYT—Sep 8th 2016); and (3) had returned from a malaria-endemic country in Africa or South East Asia within 14 days prior to admission were invited to participate in the study.

Demographics, epidemiologic, travel histories and clinical data were collected using a standardized structured questionnaire. Blood samples were taken from all enrolled patients for the detection of malaria infection using a RDT (SD BIOLINE Malaria Ag P.f/P.v), Giemsa stained blood film microscopy, and real-time polymerase chain reaction (real-time PCR). Giemsa stained blood film microscopy was performed according to the World Health Organization (WHO) standard procedure described elsewhere [17].

#### Laboratory procedures

To perform real-time PCR, total DNA was extracted from whole blood samples using QIAamp DNA Blood Mini kit (Qiagen, Cat. No.51104). Real-time PCR was performed on Real-Time PCR 7500 Fast Applied Biosystems (ABI) (ABI- USA) and targeted the 18S rRNA gene of *Plasmodium spp*. as described previously [18–20]. Selected primers and probes used as in Additional file 3: Table S1. Reaction mixture (25 µL) included master mix (10 µL) (Quantinova probe PCR kit), forward primer (10 µM; 0.5 µL), reverse primer (10 µM; 0.5 µL), probe (10 µM; 0.4 µL), purified DNA (5 µL) and nuclease-free water (3.5 µL). PCR program included 2 min of denaturation at 95 °C followed by 40 cycles of 5-s denaturation at 95 °C, 30-s annealing at 60 °C and 30-s elongation at 72 °C.

In case of recurring malaria symptoms or fever, after completing observed treatment, patients were advised to return immediately to the study team for re-evaluation. Study staff filled out a second case report form and collected blood samples for genetic testing and referred recurring cases to NIMPE. NIMPE administered treatment according to the National Malaria Treatment Guidelines. Recurring cases were not re-enrolled in the study.

### Qualitative prospective study

#### Data collection and sampling

The qualitative assessment was conducted at the NHTD and the NIMPE clinic. Qualitative data were gathered through a total of 16 in-depth interviews (IDIs) with participants of quantitative prospective study conducted between October 2017 and May 2018. The IDI guides were initially developed based on the retrospective study and on the health belief model, which was further adapted for the purpose of this study. The health belief model suggests that health behavior can be predicted by the perception of the following: risk susceptibility, risk severity, benefits of action, barriers to action, self-efficacy, and cues to action [21]. Participants were required to be in good health and willing to participate in the IDI following the description of the interview process.

### Data analysis

Data were analyzed and visualized using Tableau Desktop (Version 2018.3.3, Tableau Software, Inc., USA). Statistical analysis was performed using XLSTAT-Base statistical application for Microsoft Excel (version 2018.7, Addinsoft, France). A *Chi-square* test or *Fisher’s exact* test was used to assess the differences between proportions; the Mann–Whitney test was used to compare independent samples of patients; and *Cochran’s Q* test was used to compare multiple paired samples. *p*-value of < 0.05 was considered significant.

## Results

### Demographics

#### Quantitative retrospective strand

A total of 247 records were identified for malaria patient who returned from abroad between January 2014 and December 2016. The median age of the patients was 33.0 years (IQR 28.0–39.5 years) and the majority was male (92%). Patients predominantly self-identified as forest goers and specialized workers (130; 53%), while the remainder identified as farmers (20; 8%), technical and service workers (20; 8%) and travelers (2; 1%). The occupation of 75 (30%) patients was unknown.

**Fig. 2:**
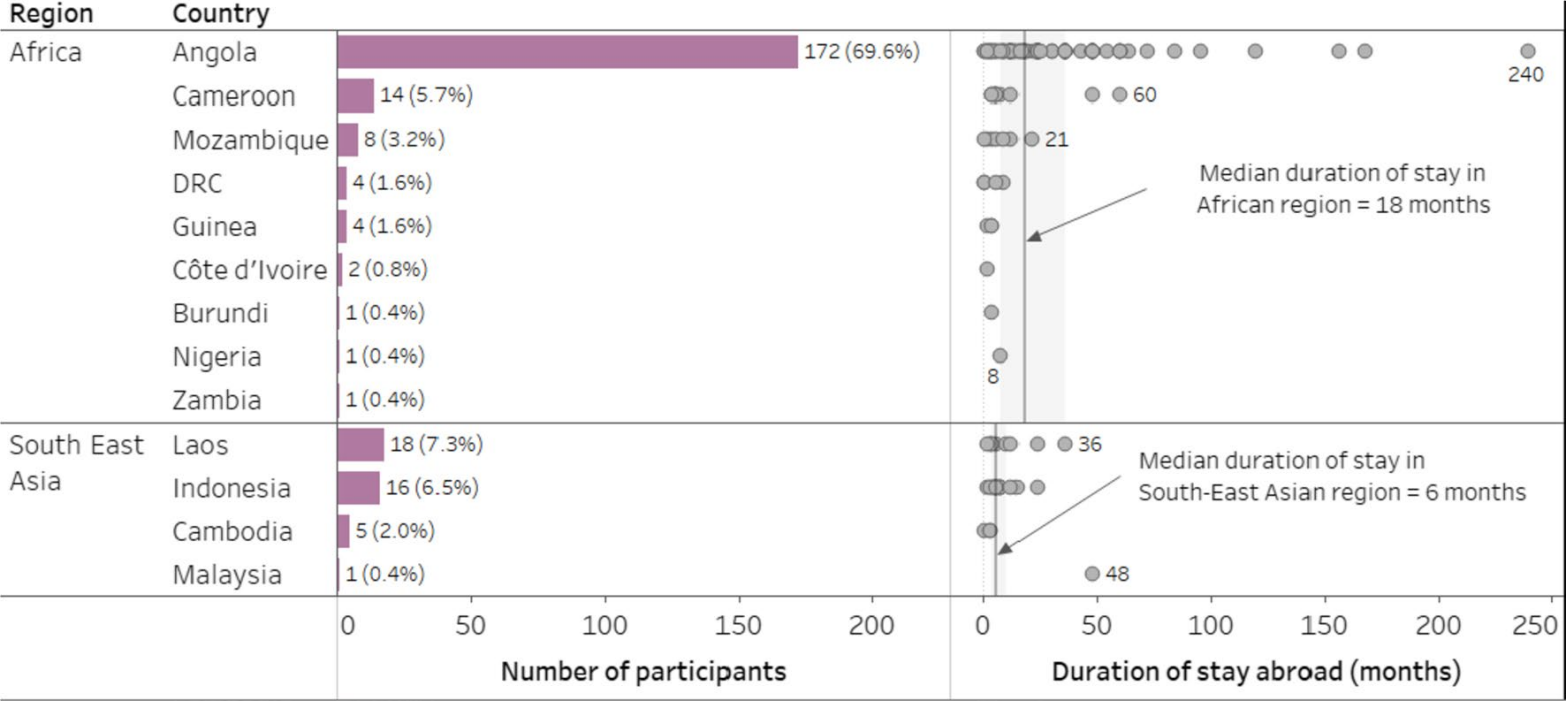
Countries visited by patients in retrospective strand and duration of staying abroad. *(Left panel: Bars indicate the total number of patients who visited a country and data labels indicate the number of patients and percentage of patients from the total number of patients. Right panel: Dots indicate the number of months spent by individual patient in a country and the data labels show the number of months abroad for selected patients. DRC* = *Democratic Republic of the Congo.)*

The majority of participants returned from African countries (n = 207; 84%) with the remainder traveling from Southeast Asian countries (n = 40; 16%; Fig. 2). The median time abroad was 13.5 months (IQR 6.0–331.5 months) for the 164 patients with data on duration of stay available. Patients stayed significantly longer in African countries (median 18.0 months; IQR 7.5–36.0 months) than in Southeast Asian countries (median 6.0 months; IQR 4.0–10.0 months; Mann–Whitney U = 2888, n_1_ = 135, n_2_ = 29, p < 0.0001 two-tailed, p̂_a>b_ = 0.738). Of all participants, 108 (44%) patients had a history of acquiring malaria abroad additionally to the current infection. The proportion of patients with a previous event was significantly lower among those who had returned from the African continent (82; 40%) than those who had returned from Southeast Asia (26; 65%), although small effect size (ES) indicated low practical significance (χ^2^ (1; 247) = 8.78, *p* = 0.003, ES Φ = 0.19).

#### Quantitative prospective strand

Travel histories, blood samples and clinical data were collected and analyzed. Excluding two participants, 38 participants were enrolled in the study, with a median age of 36 years (IQR 30.5–42 years). All participants invited to participate in the study accepted. No one refused to participate in the study.

**Fig. 3:**
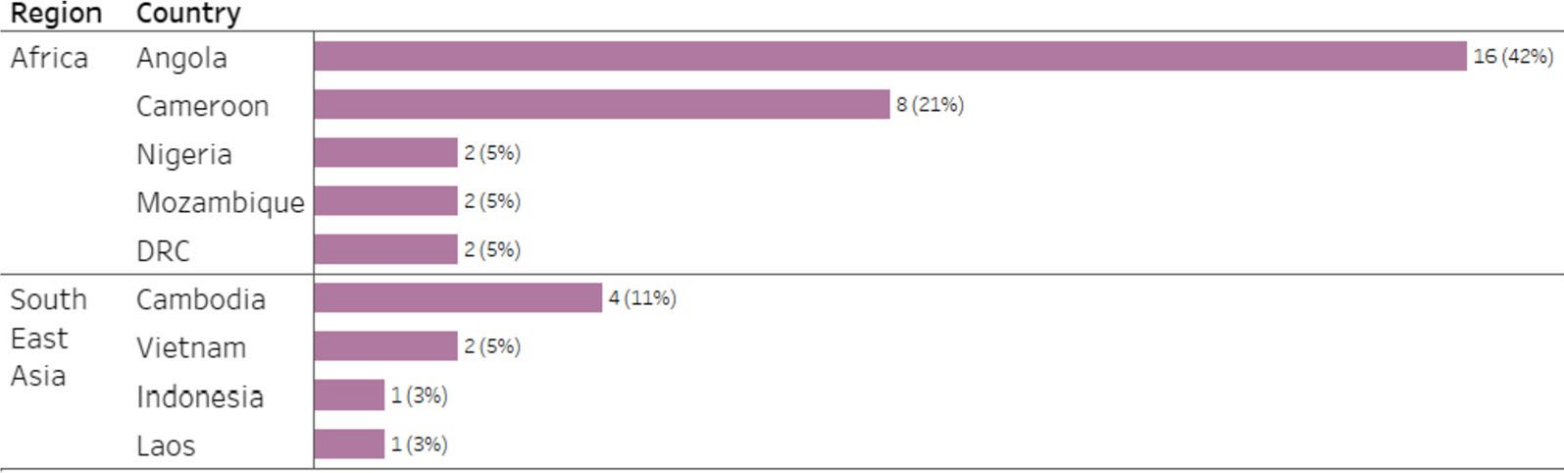
Countries visited by patients in quantitative strand. (*Data labels indicate number of patients and percentage of patients from the total number of patients visiting the region.*)

Of those enrolled, 87% (n = 33) were males and 79% (n = 30) reported having malaria at least once before, with 18% (n = 7) self-reporting more than 10 previous malaria infections. Of the enrolled patients, 78% were admitted after returning from African countries and 22% from Southeast Asian countries. Angola (n = 16; 42%) and Cameroon (n = 8; 21%) were the most common countries in which participants stayed during their last trip (Fig. 3).

**Fig. 4:**
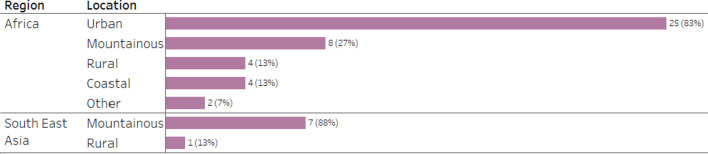
Sites of patient stay, disaggregated by region, from quantitative strand. *(Data labels indicate number of patients and percentage of patients from the total number of patients visiting the region.)*

Patients reported visits to urban (n = 25; 66%), mountainous (n = 15; 39%), rural (n = 5; 13%) and coastal (n = 4; 11%) locations, with 39% (n = 15) of patients staying exclusively at urban sites, 18% (n = 7) only at mountain sites, 13% (n = 5) visiting both urban and mountain sites, and 8% (n = 3) staying only at rural sites. Patients who traveled to Southeast Asia did not stay at urban sites (Fig. 4). Work or business (n = 31; 82%) was the most common reason for travel; other reported reasons included leisure/holiday (n = 2; 5%) and study (n = 1; 3%).

Of study participants, 58% (n = 22), 32% (n = 12), and 11% (n = 4) reported using bed nets always, frequently or not using bed nets during the latest trip, respectively. Of the 66% (n = 25) of patients who reported acquiring malaria infection during their latest trip abroad, only 8% (n = 2) reported no net use, while 60% (n = 15) used nets always and 32% (n = 8) used nets frequently.

#### Qualitative arm of prospective strand

**Fig. 5:**
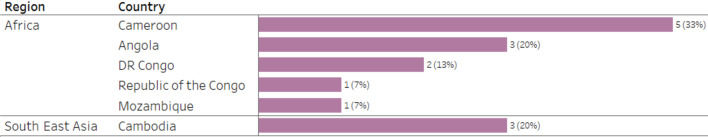
Countries where participants in qualitative arm were infected with malaria. *(Data labels show the percent of all participants infected in country and percentage of participants from the total number of participants, intensity of color corresponds with number)*

The median age for IDI participants was 37 years (IQR 32.5–41.5 years). The majority of participants (n = 15) were male (11; 73%). Most participants traveled from malaria-free regions in Northern Vietnam to Cameroon (5; 33%), Angola (4; 27%) and Cambodia (3; 20%) for employment (Fig. 5). Of 15 IDIs, 9 were employees and 6 were self-employed. Reported occupations included logging (n = 9; 60%), trading (n = 4; 27%), mining (n = 1; 7%), and technical work (n = 1; 7%). Multiple responses for occupation abroad were possible. One of the participants commented the following about his occupation: “*I cooperated with a Cambodian mine owner* *[…] to mine on his property. They provided us the land, I bought timber to build houses (wooden frames, ceilings and walls).*” (NAVY 313).

### Epidemiology

#### Quantitative retrospective strand

**Fig. 6:**
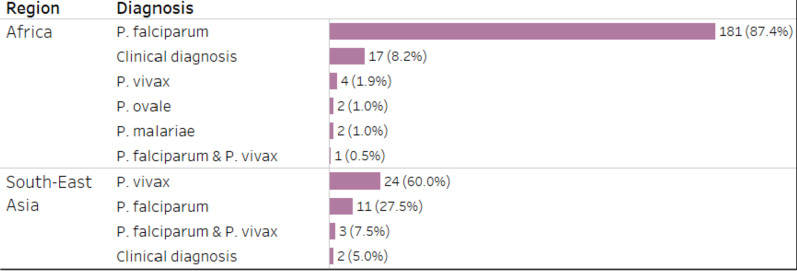
Plasmodium species diagnosed in patients from retrospective strand. *(Data labels indicate the number of patients and percentage of patients from the number of patients returned from a region.)*

*Plasmodium falciparum* comprised 192 (78%) malaria cases. Of these, 181 (94%) were diagnosed in patients who returned from Africa. While *P. falciparum* infection prevailed in travelers who returned from Africa (87% of all malaria cases acquired in Africa), infection with *P. vivax* was predominant in patients who returned from Southeast Asian countries (60% of all malaria cases acquired in Southeast Asia; Fig. 6). None of the patients who returned from Southeast Asian countries presented with *P. malariae* or *P. ovale*, while four infections with *P. malariae* or *P. ovale* (two each representing 1%, each, of all cases) were diagnosed in patients who returned from African countries.

Among 173 patients with information on duration to parasite clearance, 51 (30%) patients, including 41 (30%) patients with *P. falciparum* infection, still had measurable parasitemia by microscopy on day 3. The proportion of patients with previous history of infection was significantly lower among patients whose parasitemia cleared within 3 days or less (42%) than among patients with detectable parasitemia on day 3 (63%), although small effect size indicated weak association between time to parasite clearance and history of previous malaria infection (χ^2^ (1; 173) = 6.32, *p* = 0.012, ES Φ = 0.19). The difference in proportions of all cases with detectable parasitemia on day 3 was not significant between patients returned from the African continent and patients returned from the Southeast Asian region (χ^2^ (1; 173) = 0.29, *p* = 0.589, ES Φ = 0.04). The difference in proportions of *P. falciparum* cases with detectable parasitemia on day 3 was also not significant between patients who returned from the African continent and patients who returned from the Southeast Asian region (χ^2^ (1; 138) = 1.42, *p* = 0.234, ES Φ = 0.10).

#### Quantitative prospective strand

All patients experienced characteristic fever patterns and other malaria symptoms prior to admission and presented with a number of malaria symptoms, including fever (median temperature 38.0 °C, IQR 37.0–39.0 °C) and rapid pulse (median 85.5 beats/min, IQR 80.0–93.8 beats/min). Common malaria symptoms registered upon admission are listed in Table 1. Median duration between return from abroad and onset of symptoms was 9 days (IQR 2.5–14 days); the median duration between the onset of symptoms and admission to the hospital was 3 days (IQR 2–7 days).

**Table 1:** Number and percentage of patients from quantitative strand exhibiting malaria symptoms at admission

Malaria symptoms at admission	Frequency (%) (n = 38)	Malaria symptoms at admission	Frequency (%) (n = 38)
Persistently high fever	18 (47%)	Vomiting	5 (13%)
Typical malaria fever	17 (45%)	Accelerated respiration (> 20times/min)	3 (8%)
Prostration	12 (32%)	Diarrhea (many times)	2 (5%)
Severe headache	11 (29%)	Acute abdomen pain	1 (3%)
Jaundice	9 (24%)	Mild consciousness disorder	1 (3%)
Blue skin, pale mucosa	6 (16%)		

**Fig. 7:**
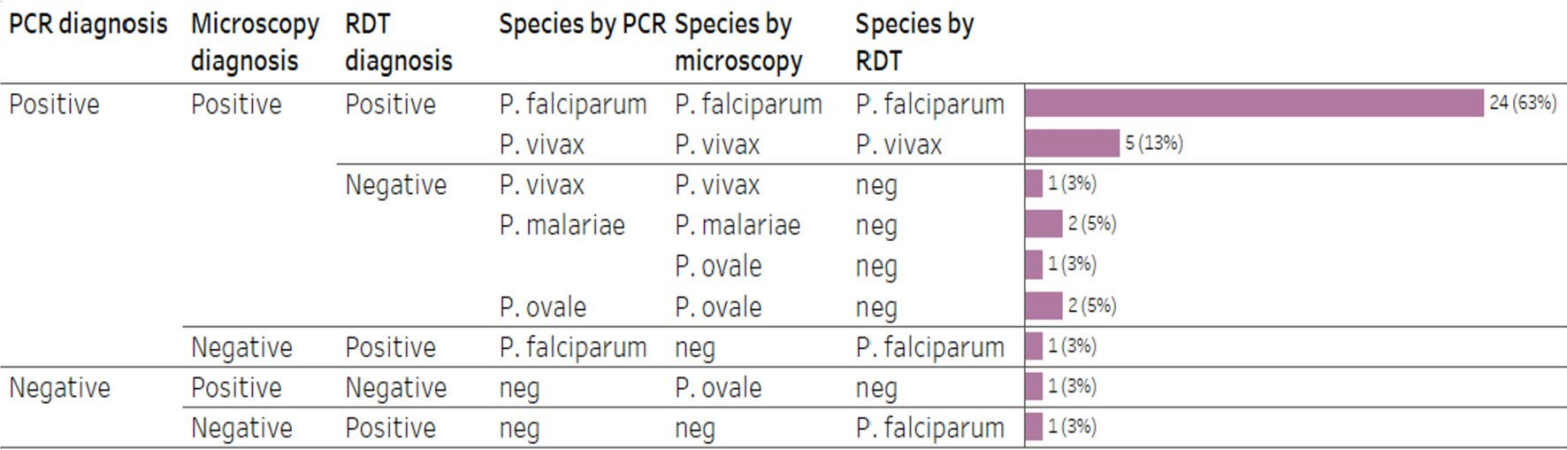
Plasmodium species diagnosed by microscopy, RDT and PCR in patients from quantitative strand. *(Data labels indicate number of patients and percentage of patients from the total number of patients.)*

All patients (38; 100%) tested positive for malaria by microscopy and/or RDT and/or PCR. Of these malaria patients, 68% (n = 26) were diagnosed with *P. falciparum,* 16% (n = 6) with *P. vivax,* 8% (n = 3) with *P. ovale,* 5% (n = 2) with *P. malariae* and 3% (n = 1) had a discrepant result of *P. malariae* by PCR and *P. ovale* by microscopy (Fig. 7)*.* Microscopy, RDT, and PCR were not significantly different in sensitivity (Cochran’s Q = 5.56, p = 0.062). In respect to specificity, all three methods detected *P. falciparum* in 24 (63%) patients and *P. vivax* in 5 (13%) patients. RDT did not detect any of the *P. malariae* and *P. ovale* cases, and one of six cases (17%) of *P. vivax*, but detected two (8%) cases of *P. falciparum,* one of which was not detected by microscopy and another by both microscopy and PCR.

**Fig. 8:**
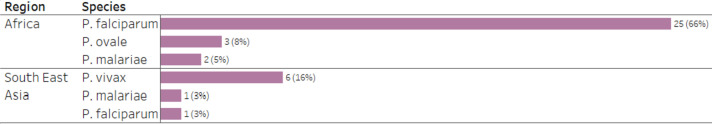
Plasmodium species diagnosed by microscopy, RDT and PCR, disaggregated by region in patients from quantitative strand. *(Data labels indicate number of patients and percentage of patients from the total number of patients.)*

Twenty-five (96%) *P. falciparum* cases and all three *P. ovale* cases originated in the African continent, while six (100%) of *P. vivax* infections were imported from the Southeast Asian region; two cases of *P. malariae* infection (as diagnosed by PCR) were imported from Africa and one from the Southeast Asia (Fig. 8). Day 3 parasite clearance rates were 58% (n = 15) and 50% (n = 3) for *P. falciparum* and *P. vivax*, respectively. The proportions of patients with previous history of infection were not significantly different between groups with parasite clearance within 3 days or less (87%) and with detectable parasitemia on day 3 (64%) (*p* = 0.215; Fisher’s exact test). There was a very weak correlation between d0 parasite density and the number of days to parasite clearance for *P. falciparum* (Sperman’s correlation analysis: *r*_*s*_ = − 0.027, *p* = 0.900) and for *P. vivax* (Sperman’s correlation analysis: *r*_*s*_ = − 0.348, *p* = 0.497). For *P. falciparum,* the association between the time to parasite clearance and the time from symptoms onset to hospital admission was also weak (Sperman’s correlation analysis: *r*_*s*_ = 0.085, *p* = 0.700).

#### Qualitative arm of prospective strand

Upon return to Vietnam, six participants (40%) were diagnosed with *P. falciparum*, four (27%) were diagnosed with *P. vivax*, and one (7%) was diagnosed with *P. ovale.* Diagnosis for four participants (27%) was not recorded. In patients who returned from the African continent, *P. falciparum* comprised 42% of malaria cases. *P. vivax* was imported from Southeast Asian countries only, while a single *P. ovale* case arrived from the African continent.

### Qualitative arm of prospective strand: knowledge of malaria

*Perceived susceptibility* IDI questions related to perceived susceptibility covered general knowledge of malaria before participants travel abroad and knowledge of the causes of malaria, specifically. Before traveling to their foreign work site, 67% (n = 10) reported having some prior knowledge of malaria, while 33% (n = 5) reported having no previous knowledge. Of 10 participants with prior knowledge, 40% (n = 4) learned about malaria on the internet while conducting research about the country they were preparing to visit, 60% (n = 6) learned from friends or acquaintances who had traveled to endemic countries, and only one participant reported being informed about malaria by a future employer. Participants who were unaware of malaria before the trip learned about malaria while in the country of their destination from co-workers (n = 1; 20%), when co-workers became sick (n = 2; 40%) or when the participant became sick (n = 1; 20%). Some discussed the challenges in finding information on malaria before departure: *“Before going to Angola, I self-searched about Angola on the internet […] no information about epidemics. I only knew about malaria after I got it in Angola.”* (NAVY 306).

Ten participants who had previous knowledge of malaria reported knowing that malaria is transmitted by mosquito bites (n = 3; 30%), that nets (n = 3; 30%) and protective clothing (n = 1; 10%) reduce risk from mosquitoes biting and that forest goers can get malaria (n = 1; 10%). Additional responses included awareness of malaria drugs (n = 2; 20%) and malaria vaccines (n = 1; 10%). Of those who reported knowledge of malaria prior to their trip, four participants (40%) reported incorrect knowledge, including not getting malaria in Vietnam “*because I eat very spicy food*”; malaria is caused by “*chemicals directly discharged in the environment*”; that “*West Africa has no malaria*”; and that exposure to contaminated water presents a risk for acquiring malaria (n = 2; 20%). Participants could provide more than one response regarding their knowledge of malaria.

*Cues to action* were covered in the IDI with the inclusion of questions on media sources accessed while abroad. Of all participants, 33% (n = 5) did not access any media. Most media accessed were Vietnamese websites, television or Facebook, due to language barriers to accessing local media (n = 7; 47%). Only 27% of participants (n = 4) reported watching local television or websites. Only one participant who accessed Vietnamese websites reported learning about malaria outbreaks from the Vietnamese community, while all four participants who watched local television received warnings about malaria and other disease outbreaks. Of all participants, 20% (n = 3) reported seeking treatment, both abroad and in Vietnam, upon advice from colleagues.

### Qualitative arm of prospective strand: transformation of knowledge about malaria

Change in knowledge about malaria was covered by questions about the cause of patient’s most recent malaria infection and malaria symptoms. With regard to the causes of patient’s malaria, the majority of participants (n = 12; 80%) were aware that their malaria was caused by mosquito bites. Of all participants, eight (53%) erroneously believed that contaminated water sources were also a cause of malaria due to presence of viruses, bacteria or chemical contamination. Participants were able to report multiple answers to this question.

Based on their own or a co-worker’s experience, all participants were able to accurately describe malaria symptoms. Among seven most commonly reported symptoms were fever or severe fever (n = 14; 93%), chills (n = 9; 60%), fatigue (n = 3; 20%), headache (n = 3; 20%), and muscle pain (n = 3; 20%) (Table 2): *“On 18 Feb, I got tired, [had] fever, headache, dizziness, but not chills. […] Brothers said I got malaria when I had symptoms of backache, diarrhea and headache.”* (NAVY 315).

**Table 2:** Knowledge about malaria symptoms

Symptoms reported	Number of participants (%) (n = 15)
Fever or severe fever	14 (93%)
Chills	9 (60%)
Fatigue	3 (20%)
Headache	3 (20%)
Muscle pain	3 (20%)
Urinary retention	2 (13%)
Vomiting	2 (13%)

Top seven symptoms of malaria listed by participants in qualitative arm

*Perceived severity* All participants considered symptoms severe enough to warrant their visit to health facility or contact of a private provider, both abroad and in Vietnam. The top three reasons for reporting to the hospital upon malaria symptoms onset were: malaria can be deadly (n = 6; 40%), the symptoms participants experienced were life-threatening (n = 11; 73%), and malaria or experienced symptoms could cause permanent damage (n = 4; 27%). The median duration between symptoms onset and reporting to the hospital was 5 days (IQR 4.0–9.5 days), and did not differ significantly between patients with prior knowledge of malaria (median 5.0 days, IQR 4.0–8.0 days) and without prior knowledge (median 6.5 days, IQR 2.5–10.5 days) (Mann–Whitney U = 15, p = 0.933).

### Qualitative arm of prospective strand: treatment seeking behavior

*Perceived Barriers to Treatment Abroad* Of patients participating in the IDIs, 12 (80%) developed malaria symptoms at their foreign work site one or more times. All 12 participants went to the hospital, a district health center or a private doctor; of the 12, two (17%) participants called a private doctor prior to hospital admission, one participant self-treated with over-the-counter medicine, and one first attempted to treat cold symptoms. None of the participants reported barriers that would prevent them from seeking health care in a foreign country. A typical response was: “*There was no difficult[y]. Health center was empty, didn’t have to line up because it was in district level. When I stayed in Angola from 2012 to 2016, my shop [was] near a provincial hospital. I was friend with some Vietnamese specialists. They said Cuban, North Korean, Russian specialists were better. Vietnamese specialist often worked in anesthesia and surgery*.” (NAVY 15). Of all participants, 33% (n = 4) who worked for a company abroad reported that the company paid for their medical treatment and provided an interpreter and transportation to the doctor’s office. Participants acknowledged the availability of anti-malarial drugs and RDTs at pharmacies in the countries they visited (n = 5; 42%), easy access to Vietnamese specialists (n = 4; 33%), and easy access to private and public hospitals (n = 3; 25%). Only one self-employed patient noted that treatment and medication were expensive, and two participants (17%) said that language barrier was an obstacle in communicating with a doctor. Some participants provided multiple answers. These experiences were found in the qualitative interviews, where a typical discussion was: “*In Cameroon, I was treated in a private hospital of indigenous people. I did not have to queue, I had a little difficulty in communication.*” (NAVY 312).

*Perceived Barriers to Treatment in Vietnam* All participants sought health care upon malaria symptoms onset in Vietnam. Of all participants, 47% (n = 7) reported going directly to the NHTD or the NIMPE clinic based on results of an internet search (n = 4; 27%) or co-worker recommendation (n = 2; 13%); 33% (n = 5) went to a district hospital, followed by transfer to the NHTD or the NIMPE clinic based on hospital (n = 3; 20%) or co-worker (n = 2; 13%) recommendations; and 20% (n = 3) reported going to a private doctor with subsequent transfer to one or two district hospitals and then the NIMPE clinic (n = 2; 13% based on hospital recommendations and n = 1; 7% based on co-worker recommendation) (Table 3). Representative comments about treatment in Vietnam were: *“I have only been treated in Vietnam.* Advantages: *Enthusiasm [of] doctors, full of medicines.* Difficulties: *Hospital at district level in Hanoi couldn’t diagnose the exact disease. […] I was introduced to NHTD by brothers working in Cameroon.” (NAVY 17)*.

**Table 3:** Health seeking behavior in Vietnam of participants in qualitative arm

Treatment seeking behavior	Source of knowledge of NIMPE	Number of participants (%) (n = 15)
Went to NHTD of NIMPE		7 (47%)
	*Co-worker recommended*	2 (13%)
	*Internet research*	4 (27%)
	*Other*	1 (7%)
Went to clinic, then NIMPE		5 (33%)
	*Co-worker recommended*	2 (13%)
	*Clinic recommended*	3 (20%)
Went to private doctor, then 2 or more hospitals, then NIMPE		3 (20%)
	*Co-worker recommended*	1 (7%)
	*First or second hospital recommended*	2 (13%)

### Qualitative arm of prospective strand: use of protective measures

Five (33%) participants reported having no knowledge of malaria prior to traveling abroad; two of them reported not having any protective measures upon arrival. Hiring companies provided protective measures for six (40%) participants and another six (40%) participants brought protective measures (hammock nets and repellents) with them. One participant was provided with a net by a friend. Protective measures provided by the companies included bed nets (n = 4), treated nets (n = 2), repellent (n = 2), air-conditioned and/or screened areas for work and lodging (n = 4). A participant in the qualitative arm explained how he prepared himself before departure: *“When I started a business in Africa, I prepared myself, including insect repellent spray and the type I buy in Vietnam. Quinine malaria drug I was given by a Korean [business] partner.” (NAVY 314).*

*Perceived Benefits* When asked about preventive measures they knew, three (20%) respondents said that “malaria cannot be prevented”; 11 (73%) named bed nets or hammock nets; five (33%) named repellent; and three (20%) mentioned the use of air-conditioning. Other responses included “clean eating”, “clean water” and “clean room” (n = 6; 40%), as well as use of anti-insect screens, mosquito traps, avoiding mosquito bites at certain times of the day and wearing long-sleeved clothes.

Of all respondents, six (40%) reported that they were not aware of treated nets. Of the six respondents who knew about treated nets, one respondent said the treated net was better than untreated, two respondents said it did not help as it was used only at night or inside where there were no mosquitoes, and three respondents reported never having used treated nets.

**Fig. 9:**
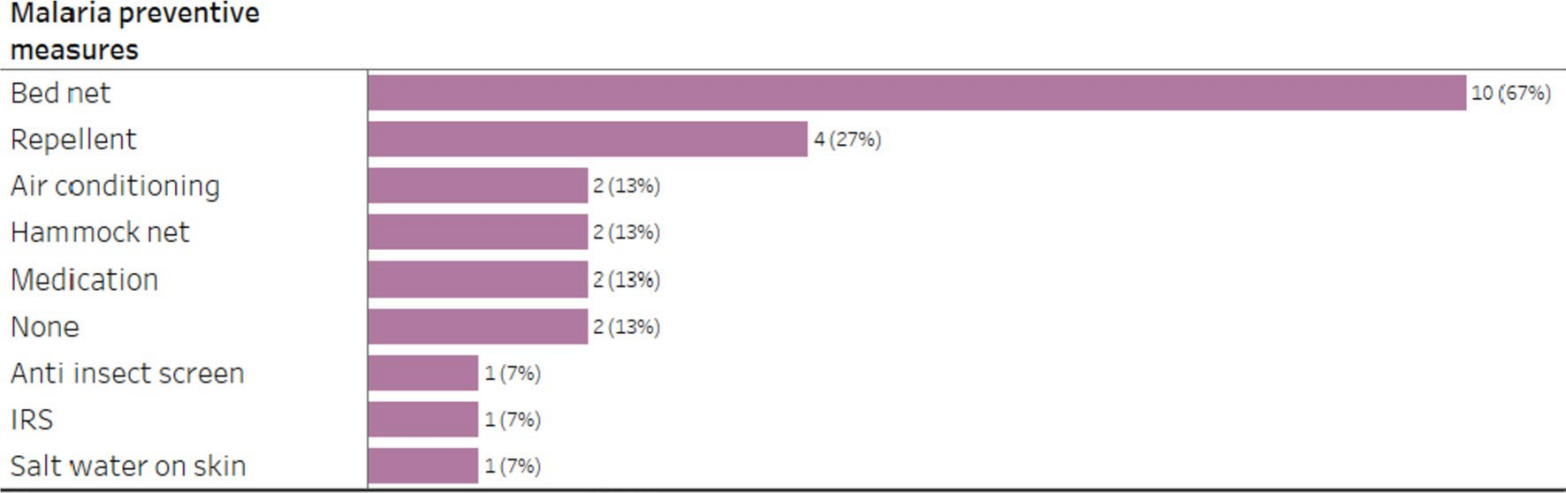
Percentage of participants in qualitative arm using protective measures

When asked about protective measures used, 10 (67%) participants reported using bed nets; four (27%) participants reported using repellents, two (13%) reported the use of medication, two (13%) reported staying in air-conditioned room, and two (13%) participants reported using hammocks. Two participants (13%) reported using no protective measures at all, with one stating that malaria is unavoidable (Fig. 9). Participants could provide more than one response regarding their use of protective measures. This statement, from an IDI with malaria patients, was typical*: “In my neighborhood, there are many mosquitoes, big mosquitoes, and I usually slept with net at night.” (NAVY 315).*

Of 10 respondents who reported frequency of preventive measures use, 50% (n = 5) always and 20% (n = 2) frequently used bed nets or hammock nets, only one participant reported frequent use of repellent (Table 4).

**Table 4:** Frequency of malaria preventive measures abroad

Malaria preventive measures	Frequency of use	Number (%) of respondents
Bed net and Hammock Net	*Always*	5 (50%)
	*Frequently*	2 (20%)
	*Sometimes*	1 (10%)
	*Rarely*	1 (10%)
Repellent	*Frequently*	1 (10%)
	*Weekly*	1 (10%)
	*During rainy season*	1 (10%)
IRS	*Monthly*	1 (10%)
Air conditioning	*Always*	1 (10%)
Filtered tap water	*Always*	1 (10%)
Salt water on skin	*Frequently*	1 (10%)

Number and percentage of participants in qualitative arm using protective measures, disaggregated by frequency of use

### Qualitative arm of prospective strand: future travel plans

**Fig. 10:**
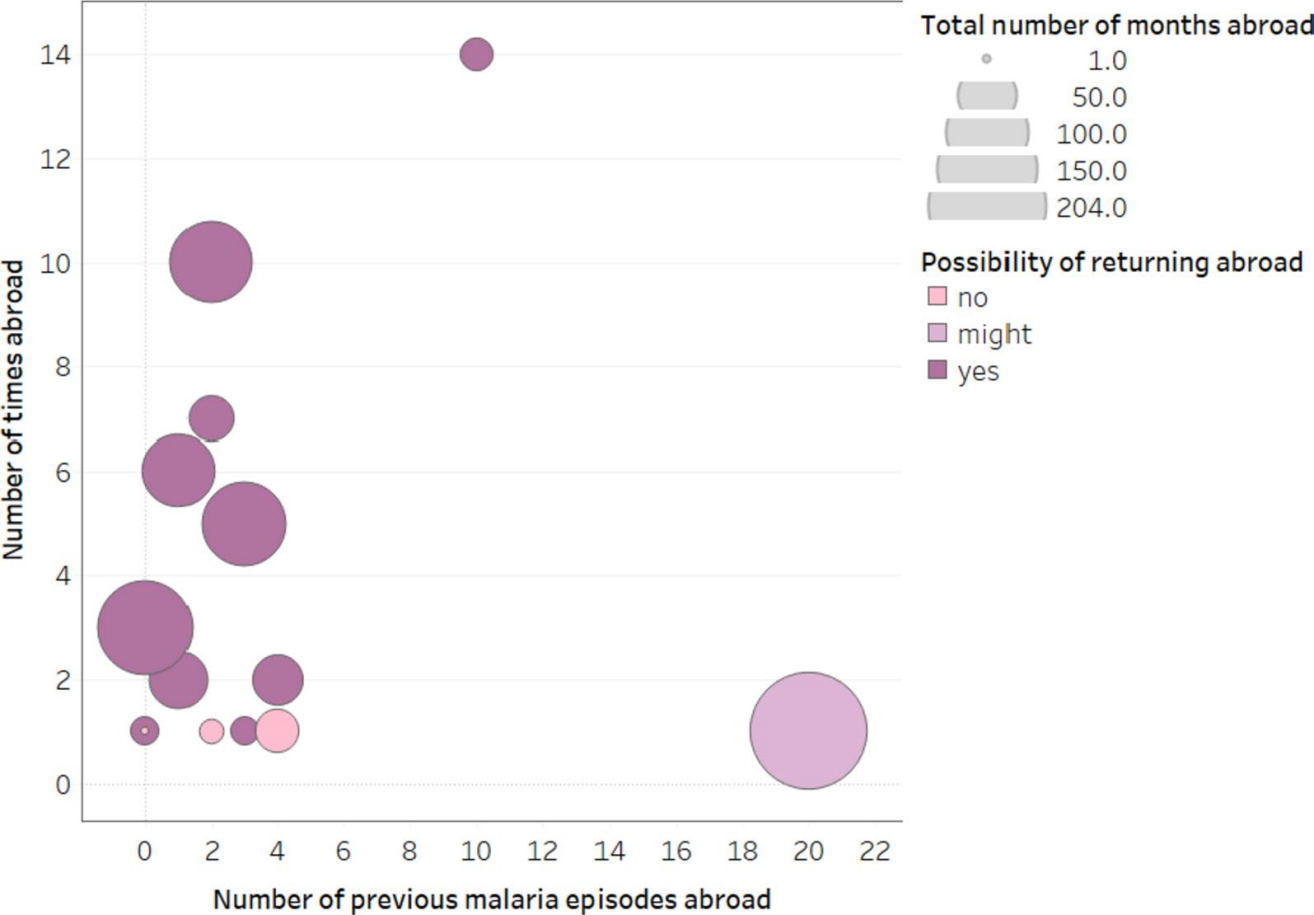
Number of times abroad and number of previous malaria episodes abroad. *(Each circle represents a participant in qualitative arm. The size of circles indicates the total number of months spent abroad and the color shows intention of participants to return abroad after treatment.)*

Only 20% (n = 3) participants indicated no intention of returning abroad, citing danger of malaria, devaluation of currency, criminal situation in a foreign country and poor health. All three of these participants traveled abroad only once, with duration of trips between 1 and 28 months. One participant who spent 17 years in Angola was not sure if he would return to Angola. The remaining 73% (n = 11) planned to return abroad soon after treatment. Of them, 64% (n = 7) have traveled abroad two or more times and have spent a total of 30 or more months abroad, while only 27% (n = 3) have traveled once for a duration of 7–12 months (Fig. 10).

## Discussion

The importation of malaria species prevalent on the African continent or drug-resistant malaria from neighboring Southeast Asian countries may present a threat to malaria-naïve populations in Vietnam and necessitates consideration in future revisions of the national malaria elimination strategy.

This study demonstrated that international laborers returning to Vietnam were predominately young men working in occupations, such as logging, that put them at higher risk for malaria infection, most commonly in the African continent. The majority of participants stayed abroad for prolonged periods of time and had previously acquired malaria abroad before the case with which they presented for care at one of the study sites. The majority of patients enrolled in the study presented with *P. falciparum* and *P. vivax*. *P. falciparum* was the dominant species overall and in patients who returned from the African continent, while *P. vivax* was predominately diagnosed in patients returning from Southeast Asia. However, diagnosis of several cases of *P. ovale* and *P. malariae* that originated primarily in the African continent may present a unique challenge for NMCP focused on the elimination of endemic *P. falciparum* and *P. vivax*. In the quantitative prospective strand, the proportion of *P. ovale* and *P. malariae* was as high as 8% for each species and all but one *P. malariae* case originated in the African continent.

*Plasmodium falciparum* day 3 parasite clearance rates were 70% for the quantitative retrospective strand (data not shown) and 58% for the quantitative prospective strand, which is below the 90% threshold for suspected artemisinin resistance, as defined by the WHO [3]. Although the presence of drug resistant *P. falciparum* strains was not investigated, other findings, such as poor correlation between d0 parasite count and the number of days to parasite clearance, as well as correlation between the time to parasite clearance, have been associated with the development of drug resistance. However, other factors not measured in this study cannot be ruled out, such as delayed health seeking behavior upon return from abroad and unsupervised adherence to malaria treatment.

The study did not provide strong evidence that previous malaria infections contributed to prolonged parasite clearance in either the quantitative retrospective or quantitative prospective strands. The study also did not show the difference in day 3 overall parasite clearance rates and *P. falciparum* clearance rates between patients returning from African and Southeast Asian countries.

The study results demonstrated gaps in knowledge about malaria and misconceptions regarding malaria transmission and appropriate protective measures. Although many participants reported not knowing of malaria before traveling abroad, they reported learning about malaria on the internet or while living abroad. Many participants cited mosquito bites as the source of malaria infection, but many participants also cited erroneous sources of malaria infection, including contaminated water. Overall, up to 89% of patients in the quantitative prospective strand and up to 70% in the qualitative arm of the prospective strand reported using protective measures always or frequently while abroad. Of interest, patients’ employers reportedly provided personal protective measures and work environments that could potentially limit malaria transmission, for example, office spaces with screens and/or air-conditioning. Having used protective measures yet acquiring malaria led several patients to conclude that “*malaria cannot be prevented*”. Thus, additional investigation into appropriate malaria prevention and intervention strategies targeted towards international laborers, both at pre-departure and upon return is recommended.

Overall, while abroad, patients did not have difficulties accessing treatment. In several cases, employers also assisted financially and logistically with the access to medical assistance. However, some patients who self-financed their treatment also noted high costs of anti-malarial drugs.

Patients in the qualitative arm indicated that knowledge about malaria, including malaria symptoms, gained abroad was the reason for seeking medical help immediately after the onset of malaria symptoms. Indeed, the median duration between symptoms onset and admission to the hospital was 3 days (IQR 2–7 days) for the entire quantitative prospective strand and 5 days (IQR 4.0–9.5 days) for the qualitative arm of the prospective strand and did not differ significantly between patients in qualitative arm with and without prior knowledge about malaria. Significantly, a barrier which prevented some patients from getting immediate medical assistance upon return to Vietnam was the concentration of malaria expertise and necessary equipment, diagnostics and medicines in specialized malaria clinics. Those patients who did not know about specialized clinics in Vietnam and initially reported to district hospitals subsequently sought or were referred to specialized clinics. Therefore, coordination between specialized clinics and district hospitals could improve access to care for imported malaria patients returning from abroad.

Study limitations included small sample size for the quantitative prospective strand and its qualitative arm. During the study period, there were new regulations regarding foreign workers working in Angola. Since Angola was the main country for work for Vietnamese workers, the number of participants in this study was smaller than planned in the study protocol. However, we believe that the findings are still very relevant to inform the national malaria elimination strategy in Vietnam and other malaria eliminating countries.

Additionally, although the study provided deeper insight into the demographics of and malaria epidemiology in international laborers, some of the study results, e.g., time to parasite clearance and dependence between parasite clearance and history of malaria infections, should be interpreted with caution, given the aforementioned lack of single standardized treatment protocol in the quantitative retrospective and quantitative prospective strands and the small sample size of the qualitative arm.

When considering the rate of parasite clearance, the data should be interpreted carefully, as there was not a single controlled standard of care. Specifically, records on medication used for treatment was not available for all the patients in the retrospective strand, while available records indicated use of diverse treatment protocols.

Not all patients participated in the qualitative strand of this study. Some patients were too sick to participate in the IDIs while others had already been discharged from the hospital before the interviewer arrived. The qualitative interviewer was different from the quantitative interviewer because the qualitative interviewer had received training on qualitative methodologies. This could have introduced selection bias into the study since these patients might have had different perspective that were not represented in the findings of this study. However, this shows the reality of conducting a study in a real life setting and the findings are still very relevant to inform pre-departure, upon arrival and return interventions for Vietnamese migrant workers.

## Conclusion

Improved understanding of global linkages, especially through international labor, and their role in re-introducing or sustaining ongoing transmission is crucial for developing and sustaining effective malaria elimination strategies. Interventions aimed at the emerging high-risk population of international laborers should cover all key stages from pre-departure, to time abroad, to return to Vietnam. Although the study sample size was small, there was some evidence that patients became educated on malaria transmission, symptoms and treatment at their place of work abroad and took the signs of infection seriously. Most participants reported to the hospital immediately upon their return. However, some, especially those with *P. vivax* infection, did not. Therefore, elimination efforts should not rely on international travelers becoming self-educated. Education for international laborers before their departure is an important factor in malaria elimination. While many participants reported use of bed nets, further attention should be given to promoting the use of additional methods of prevention and prophylaxis while in-country. Finally, consideration for coordination between specialized clinics and district hospitals in areas with returning workers should be considered to provide malaria expertise and supplies closer to the initial point of care.

## Supplementary Information

**Additional file 1.** **TOTO-IDI Guideline**: In-depth interview guideline.

**Additional file 2.** **Final-CRF-EN**: Case report form.

**Additional file 3.** **Table S1**: Primers and probes used for laboratory procedures.

## Data Availability

The datasets used and/or analyzed during the current study are available from the corresponding author on reasonable request.

## Abbreviations

AAA: American Anthropological Association
IDIs: In-depth interviews
IQR: Inter-quintile range
IRB: Institutional Review Board
HRPO: Navy Human Research Protection Office
NIMPE: Vietnam National Institute of Malariology, Parasitology and Entomology
NMCP: The Vietnam National Malaria Control Program
NHTD: National Hospital of Tropical Disease
RDT: Rapid diagnostic test
real-time PCR: Real-time polymerase chain reaction
WHO: World Health Organization
