# Supplementary material for: Understanding the epidemiology, clinical characteristics, knowledge and barriers to treatment and prevention of malaria among returning international laborers in northern Vietnam: a mixed-methods study
Source: BMC Infect Dis. 2022 May 13;22:460. doi: 10.1186/s12879-022-07322-5 (PMC9102356; doi:10.1186/s12879-022-07322-5)
Supplement: Supplementary file 1 — Additional file 1. TOTO-IDI Guideline: In-depth interview guideline. [file 12879_2022_7322_MOESM1_ESM.docx]

**TOPIC GUIDE – IN DEPTH INTERVIEW (IDI)**

**TOTO QUALITATIVE COMPONENT**

Introduce yourself and organization:

Introduce topic (length of FGD approx. 45 mins, confidentiality, and informed consent)

Objective of the study: *to understand migration patterns malaria prevention and treatment practices among Vietnamese who acquire malaria abroad.*

IDI facilitator: ____________________ ID note taker: ____________________

Date: ____________________ Time: ____________________ Study code: ____________________

| 1)Name: |  |  |
| --- | --- | --- |
| 2)Phone number: | 3)Year of birth: | 4)Gender: |
| 5)Got malaria from (country/s abroad): | Date of return |  |
| 6)Province/s of residence in Vietnam: |  |  |
| 7)Occupation/s in Vietnam: |  |  |
| 8)Occupation/s abroad: |  |  |
| 9)Diagnosis (type of parasite):  Date of diagnosis |  |  |

**QUESTIONS / PROBES**

| **1.** | **MIGRATION PATTERNS** |
| --- | --- |
| **1.1.** | What was the purpose/s of you going to ________ (the country where you come back from)? |
| **1.2.** | What did you do in ________ (the country where you come back from)? |
| **1.3.** | Tell me about the processes you when through when you first decided to go to ________ (the country where you come back from)?  PROMPTS:  Where did you get information from? /what is the company/agent?  What was your preparation process?  What was the registration process? |
| **1.4.** | How many times have you been to ________ (the country where you come back from)?  **AND** How long did you usually stay on each trip? |
| **1.5.** | Describe where(s) you stay while at ________ (the country where you come back from)?  PROMPTS:   \| Area? \| Rural/ urban \| \| --- \| --- \| \| Type housing? \| Building/ department/ tent/ \| \| Material? \| Concrete/ cement/ wood/ \| \| Ownership? \| Dormitory/rent/  Air conditioned? Screened? \| \|  \|  \| |
| **1.6** | Describe the place(s) where you work while at ________ (the country where you come back from)?  PROMPTS:   \| Area? \| Rural/ urban \| \| --- \| --- \| \| Type housing? \| Building/ department/ tent/ \| \| Material? \| Concrete/ cement/ wood/ \| \| Ownership? \| Dormitory/rent/ \| |
|  | |
| **2.** | **MALARIA EDUCATION** |
|  | **PRE-DEPARTURE** |
| **2.1.** | Tell us about what you knew about malaria and malaria prevention in Vietnam **before going abroad**?  PROMPTS:   \| By whom? \| From where? \| \| --- \| --- \| \| By how? \| What’s source? \| |
| **2.2.** | Did anybody provide you with a bed net, repellent, or any preventive measure **before going abroad**?  **If YES,** who? and where? |
|  | **ON-ARRIVAL** |
| **2.3.** | What media did you listen to or watch when you were in ______ (the country where you come back from)?  If **YES**, which type of media?  If **NO**, what were the difficulties? Was it at your place of residence? Where you slept? |
| **2.4.** | Tell us about anything you learnt about malaria and malaria prevention in **________ (the country where you come back from)**?  PROMPTS:   \| By whom? \| From where? \| \| --- \| --- \| \| By how? \| What’s source? \| \|  \|  \| |
| **2.5.** | What malaria prevention materials or products did you have in **________ (the country where you come back from)**?  If **YES**, who? and where? |
|  | |
| **3.** | **TREATMENT SEEKING BEHAVIORS AND BARRIERS TO TREATMENT** |
| **3.1.** | Tell us about what you did for when you first felt unwell /or thought you may have malaria this last time? |
| **3.2.** | What helped or what made it hard for you to obtain treatment for your case of malaria in ______________ (the country where they come back from)?  PROMPTS: language/ stock out/migrant registration issues factors/ didn’t know where to go |
| **3.3.** | So what did you do for your treatment when you came back here is Vietnam?  PROMPTS How did you get to know about this hospital **in Vietnam**? |
|  | |
| **4.** | **ABROAD – KNOWLEDGE ABOUT MALARIA (causes, prevention, and treatment)** |
| **4.1.** | What do you think caused you to get malaria when you were in _________? |
| **4.2.** | What made you think the malaria was serious enough to seek treatment?  PROMOT What signs and symptoms made you decide the malaria is serious? |
| **4.3.** | Before you went to and whilst you were in __________(the country where you come from) what methods did you know about to prevent you getting malaria ?  PROMPT How about mosquito/bed nets. Do you think there is a difference between insecticide treated nets versus non-treated nets?  If **YES**, how would insecticide treated nets benefit you? |
|  | |
| **5.** | **CLOSING** |
| **5.1** | Will you be going back to __________________? **AND** Why? |
| **5.2** | What’s next after you come back from________ (the country where you come back from)? |

**CLOSURE**

- Review any patterns or themes that emerged during the interview.
- Ask: Do you have anything else you want to add to the discussion?
- Thank the participant for their time and assure him/her that their observations and comments have been very useful.

**END In depth interview**

Thank respondent for taking the time to participate in the in-depth interview
