# Supplementary material for: Understanding the epidemiology, clinical characteristics, knowledge and barriers to treatment and prevention of malaria among returning international laborers in northern Vietnam: a mixed-methods study
Source: BMC Infect Dis. 2022 May 13;22:460. doi: 10.1186/s12879-022-07322-5 (PMC9102356; doi:10.1186/s12879-022-07322-5)
Supplement: Supplementary file 2 — Additional file 2. Final-CRF-EN: Case report form. [file 12879_2022_7322_MOESM2_ESM.docx]

| 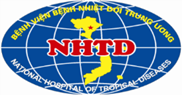   \| **MINISTRY OF HEALTH NATIONAL HOSPITAL FOR TROPICAL DISEASES CLINICAL LABORATORY – TEL: 04.6278 2034 (*Appendix* 02)** \| \| --- \| | | | | | | | | | | | | | | | | |
| --- | --- | --- | --- | --- | --- | --- | --- | --- | --- | --- | --- | --- | --- | --- | --- | --- | --- |
| **CASE REPORT FORM**   \| **Consent:** \| **Site:** \| **Date** \| \| --- \| --- \| --- \| \|  \|  \|  \| | | | | | | | | | | | | | | | | |
| **GENERAL INFORMATION** | | | | | | | | | | | | | | |  | |
| **No.** | **Contents** | | **Answers** | | | | **Code** | | | **Note** | | | | |  | |
| A1. | Study code | | Navy……………………..….. | | | |  | | |  | | | | |  | |
| A2. | Patient's hospital record code | | ………………………….……. | | | |  | | |  | | | | |  | |
| A3. | Date of birth (dd/mm/yy): | | …...../…...../...…… | | | |  | | |  | | | | |  | |
| A4. | Gender: | | Female | | | | 1 | | |  | | | | |  | |
|  |  | | Male | | | | 2 | | |  | | | | |  | |
| A5. | Occupation | | Famer | | | | 1 | | |  | | | | |  | |
|  |  | | Worker | | | | 2 | | |  | | | | |  | |
|  |  | | Official/Civil servant | | | | 3 | | |  | | | | |  | |
|  |  | | Trader | | | | 4 | | |  | | | | |  | |
|  |  | | Retired | | | | 5 | | |  | | | | |  | |
|  |  | | Housewife | | | | 6 | | |  | | | | |  | |
|  |  | | Other (…...……….....….) | | | | 7 | | |  | | | | |  | |
| A6. | Address | | ………………………….……. | | | |  | | |  | | | | |  | |
| A7 | District | | ………………………….……. | | | |  | | |  | | | | |  | |
| A8 | Province | | ………………………….……. | | | |  | | |  | | | | |  | |
| A9 | TEL. | | ………………………….……. | | | |  | | |  | | | | |  | |
| A10. | Admission date (dd/mm/yy): | | …...../…...../...…… | | | |  | | |  | | | | |  | |
| **EPIDEMIC** | | | | | | | | | | | | | | |  | |
| **No.** | **Contents** | | **Answers** | | | | | | **Code** | | | **Note** | |  | | |
| B1 | How many times you have got malaria in the past | | .................. time | | | | | |  | | | **0 🡪B5** | |  | | |
| B2 | When was your last malaria episode (dd/mm/yy)? | | …...../…...../...…… | | | | | |  | | |  | |  | | |
| B3 | Where were you diagnosed in your last malaria episode? | | Public hospital | | | | | | 1 | | |  | |  | | |
|  |  |  | Private hospital | | | | | | 2 | | |  | |  | | |
|  |  |  | Personal clinics | | | | | | 3 | | |  | |  | | |
| B4 | In which country were you diagnosed? | | Angola | | | | | | 1 | | |  | |  | | |
|  |  |  | Cameroon | | | | | | 2 | | |  | |  | | |
|  |  |  | Congo | | | | | | 3 | | |  | |  | | |
|  |  |  | Guinea | | | | | | 4 | | |  | |  | | |
|  |  |  | Mozambique | | | | | | 5 | | |  | |  | | |
|  |  |  | South Africa | | | | | | 6 | | |  | |  | | |
|  |  |  | Nigeria | | | | | | 7 | | |  | |  | | |
|  |  |  | Laos | | | | | | 8 | | |  | |  | | |
|  |  |  | Cambodia | | | | | | 9 | | |  | |  | | |
|  |  |  | Viet Nam | | | | | | 10 | | |  | |  | | |
|  |  |  | Indonesia | | | | | | 11 | | |  | |  | | |
|  |  |  | Malaysia | | | | | | 12 | | |  | |  | | |
|  |  |  | Other (……….……….....….) | | | | | | 13 | | |  | |  | | |
| B5 | Have you ever been away left your place of residence? | | Never | | | | | | 1 | | | 🡪 C1 | |  | | |
|  |  |  | Yes | | | | | | 2 | | |  | |  | | |
| B6 | When was the last time you were away your place of residence (dd/mm/yy)? | | …...../…...../...…… | | | | | |  | | |  | |  | | |
| B7 | Which country did you go to? | | Angola | | | | | | 1 | | |  | |  | | |
|  |  |  | Cameroon | | | | | | 2 | | |  | |  | | |
|  |  |  | Congo | | | | | | 3 | | |  | |  | | |
|  |  |  | Guinea | | | | | | 4 | | |  | |  | | |
|  |  |  | Mozambique | | | | | | 5 | | |  | |  | | |
|  |  |  | Nam Phi | | | | | | 6 | | |  | |  | | |
|  |  |  | Nigeria | | | | | | 7 | | |  | |  | | |
|  |  |  | Laos | | | | | | 8 | | |  | |  | | |
|  |  |  | Cambodia | | | | | | 9 | | |  | |  | | |
|  |  |  | Viet Nam | | | | | | 10 | | |  | |  | | |
|  |  |  | Indonesia | | | | | | 11 | | |  | |  | | |
|  |  |  | Malaysia | | | | | | 12 | | |  | |  | | |
|  |  |  | Others (……….……….....….) | | | | | | 11 | | |  | |  | | |
| B8 | Which province did you go to? | | ……….……….....…. | | | | | |  | | |  | |  | | |
| B9 | When did you return Vietnam/your place of residence (dd/mm/yy)? | | …...../…...../...…… | | | | | |  | | |  | |  | | |
| B10 | What was your purpose to go there? | | Travel | | | | | | 1 | | |  | |  | | |
|  |  |  | Business (Specify……………..) | | | | | | 2 | | |  | |  | | |
|  |  |  | Working (Specify……………..) | | | | | | 3 | | |  | |  | | |
|  |  |  | Study | | | | | | 4 | | |  | |  | | |
|  |  |  | Others (……….……….....….) | | | | | | 5 | | |  | |  | | |
| B11 | Characteristics of this place?  **(Multiple choices question)** | | urban | | | | | | 1 | | |  | |  | | |
|  |  |  | rural | | | | | | 2 | | |  | |  | | |
|  |  |  | coast | | | | | | 3 | | |  | |  | | |
|  |  |  | mountainous | | | | | | 4 | | |  | |  | | |
| B12 | How frequently did you use bed nets during that time? | | No (0-30%) | | | | | | 1 | | |  | |  | | |
|  |  |  | Sometimes (30-70%) | | | | | | 2 | | |  | |  | | |
|  |  |  | Frequently (71-90%) | | | | | | 3 | | |  | |  | | |
|  |  |  | Always (91-100%) | | | | | | 4 | | |  | |  | | |
| B13 | During this trip, did you contract malaria? | | Yes | | | | | | 1 | | |  | |  | | |
|  |  |  | No | | | | | | 2 | | |  | |  | | |
| B14 | Beside this trip, in the last 5 years, have you been aboard? | | Yes | | | | | | 1 | | |  | |  | | |
|  |  |  | No | | | | | | 2 | | | 🡪 C1 | |  | | |
|  | **Please provide in details for all your leaves**  **(Chose the number corresponding to each leaving time)** | | | | | | | | | | | | | | | |
|  |  |  | |  | **1st time** | **2^nd^ time** | | **3^rd^ time** | | | **4^th^ time** | | **5^th^ time** | | | |
| B15 | Which country you went? | Angola | | 1 |  |  | |  | | |  | |  | | | |
|  |  | Cameroon | | 2 |  |  | |  | | |  | |  | | | |
|  |  | Congo | | 3 |  |  | |  | | |  | |  | | | |
|  |  | Guinea | | 4 |  |  | |  | | |  | |  | | | |
|  |  | Mozambique | | 5 |  |  | |  | | |  | |  | | | |
|  |  | South Africa | | 6 |  |  | |  | | |  | |  | | | |
|  |  | Nigeria | | 7 |  |  | |  | | |  | |  | | | |
|  |  | Laos | | 8 |  |  | |  | | |  | |  | | | |
|  |  | Cambodia | | 9 |  |  | |  | | |  | |  | | | |
|  |  | Indonesia | | 10 |  |  | |  | | |  | |  | | | |
|  |  | Malaysia | | 11 |  |  | |  | | |  | |  | | | |
|  |  | Other(………..) | | 12 |  |  | |  | | |  | |  | | | |
| B16 | How many months from now? | ………. Month | |  |  |  | |  | | |  | |  | | | |
| B17 | What was your purpose to go there? | Travel | | 1 |  |  | |  | | |  | |  | | | |
|  |  | Business **(specify)** | | 2 |  |  | |  | | |  | |  | | | |
|  |  | Working **(specify)** | | 3 |  |  | |  | | |  | |  | | | |
|  |  | Study | | 4 |  |  | |  | | |  | |  | | | |
|  |  | Other(………..) | | 5 |  |  | |  | | |  | |  | | | |
| B18 | How long did you stay there? | ………days | |  |  |  | |  | | |  | |  | | | |
| B19 | How frequently did you use bed nets during that time? | No (0-30%) | | 1 |  |  | |  | | |  | |  | | | |
|  |  | Sometimes (30-70%) | | 2 |  |  | |  | | |  | |  | | | |
|  |  | Frequently (71-90%) | | 3 |  |  | |  | | |  | |  | | | |
|  |  | Always (91-100%) | | 4 |  |  | |  | | |  | |  | | | |
| B20 | During this time, did you contract malaria? | Yes | | 1 |  |  | |  | | |  | |  | | | |
|  |  | No | | 2 |  |  | |  | | |  | |  | | | |
| **PATIENT HOSPITAL RECORD** | | | | | | | | | | | | |  | | | |
| **I. INFORMATION AT THE ADMISSION DATE** | | | | | | | | | | | | |  | | | |
| **No.** | **Contents** | | **Answers** | | | | | **Code** | | | | **Note** | |  | | |
| C1. | When did the fever start (dd/mm/yy)? | | …...../…...../...…… | | | | |  | | | |  | |  | | |
| C2 | Temperature | | ………….…………….... °C | | | | |  | | | |  | |  | | |
| C3 | Each fever lasted for | | ……………………....….hours | | | | |  | | | |  | |  | | |
| C4 | Intervals between fevers | | ……………………....….hours | | | | |  | | | |  | |  | | |
| C5 | Have you taken any medicines before medical examination? | | No | | | | | 1 | | | | - D1 | |  | | |
|  |  |  | Yes | | | | | 2 | | | |  | |  | | |
| C6 | Which medications did you take before medical examination? | | Arterakine | | | | | 1 | | | |  | |  | | |
|  |  |  | Chloroquin | | | | | 2 | | | |  | |  | | |
|  |  | | Primaquin | | | | | 3 | | | |  | |  | | |
|  |  | | Quinin sulfate | | | | | 4 | | | |  | |  | | |
|  |  | | Quinin hydrochloride | | | | | 5 | | | |  | |  | | |
|  |  | | Clindamycin | | | | | 6 | | | |  | |  | | |
|  |  | | Doxycyclin | | | | | 7 | | | |  | |  | | |
|  |  | | Artesunate | | | | | 8 | | | |  | |  | | |
|  |  | | Mefloquin | | | | | 9 | | | |  | |  | | |
|  |  | | Others (…...……….....….) | | | | | 10 | | | |  | |  | | |
| **II. Examination: INFORMATION AT ADMISSION DATE** | | | | | | | | | | | | |  | | | |
| **No.** | **Contents** | | **Answers** | | | | | | **Code** | | | **Note** | |  | | |
| D1. | Check vital signs: | |  | | | | | |  | | |  | |  | | |
| D2 | Pulse | | ………….….times/min | | | | | |  | | |  | |  | | |
| D3 | Breathing rate | | ………….….times/min | | | | | |  | | |  | |  | | |
| D4 | Blood pressure (max/min) | | ………………….….…mmHg | | | | | |  | | |  | |  | | |
| D5 | Temperature | | ………….…………….... °C | | | | | |  | | |  | |  | | |
| D6 | Symptom | | Typical malaria fever | | | | | | 1 | | |  | |  | | |
|  |  | | Persistently high fever | | | | | | 2 | | |  | |  | | |
|  |  | | Prostration | | | | | | 3 | | |  | |  | | |
|  |  | | Severe headache | | | | | | 4 | | |  | |  | | |
|  |  | | Acute abdomen pain | | | | | | 5 | | |  | |  | | |
|  |  | | Vomiting | | | | | | 6 | | |  | |  | | |
|  |  | | Diarrhea (many times) | | | | | | 7 | | |  | |  | | |
|  |  | | Gastrointestinal Hemorrhage | | | | | | 8 | | |  | |  | | |
|  |  | | Hemorrhage under the skin | | | | | | 9 | | |  | |  | | |
|  |  | | Blue skin, pale mucosa | | | | | | 10 | | |  | |  | | |
|  |  | | Jaundice | | | | | | 11 | | |  | |  | | |
|  |  | | Oliguria | | | | | | 12 | | |  | |  | | |
|  |  | | Anuretic | | | | | | 13 | | |  | |  | | |
|  |  | | Acute Kidney failure | | | | | | 14 | | |  | |  | | |
|  |  | | Accelerated respiration (>20times/min) | | | | | | 15 | | |  | |  | | |
|  |  | | Pulmonary edema | | | | | | 16 | | |  | |  | | |
|  |  | | Moist rale in 2 lung's bottom | | | | | | 17 | | |  | |  | | |
|  |  | | Mild consciousness disorder | | | | | | 18 | | |  | |  | | |
|  |  | | Coma | | | | | | 19 | | |  | |  | | |
|  |  | | Convulsion >2times/24 hours | | | | | | 20 | | |  | |  | | |
| D7 | Blood film examination - Giemsa stained | | Positive | | | | | | 1 | | |  | |  | | |
|  |  |  | Negative | | | | | | 2 | | |  | |  | | |
| D8 | Species | | *P. falciparum* | | | | | | 1 | | |  | |  | | |
|  |  | | *P. vivax* | | | | | | 2 | | |  | |  | | |
|  |  | | *P. malariae* | | | | | | 3 | | |  | |  | | |
|  |  | | *P. ovale* | | | | | | 4 | | |  | |  | | |
|  |  | | *P. knowlesi* | | | | | | 5 | | |  | |  | | |
| D9 | State | | Ring forms or Tropozoites | | | | | | 1 | | |  | |  | | |
|  |  | | Gametocytes | | | | | | 2 | | |  | |  | | |
|  |  | | Schizonts | | | | | | 3 | | |  | |  | | |
| D10 | Malaria density | | …………...parasites/mm3 | | | | | |  | | |  | |  | | |
| D11 | Result of rapid diagnostic test | | Positive | | | | | | 1 | | |  | |  | | |
|  |  |  | Negative | | | | | | 2 | | |  | |  | | |
| D12 | Species | | *P. falciparum* | | | | | | 1 | | |  | |  | | |
|  |  | | *P. vivax* | | | | | | 2 | | |  | |  | | |
| **III. Indications for treatment:** | | | | | | | | | | | | |  | | | |
| **No.** | **Contents** | | **Medication** | | | | | | **mg** | | | **Duration of treatment (days)** | | | | |
| E1. | Medicines and dosage | | Arterakine | | | | | |  | | |  | |  | | |
|  |  | | Chloroquin | | | | | |  | | |  | |  | | |
|  |  | | Primaquin | | | | | |  | | |  | |  | | |
|  |  | | Quinin sulfate | | | | | |  | | |  | |  | | |
|  |  | | Quinin hydrochloride | | | | | |  | | |  | |  | | |
|  |  | | Clindamycin | | | | | |  | | |  | |  | | |
|  |  | | Doxycyclin | | | | | |  | | |  | |  | | |
|  |  | | Artesunate | | | | | |  | | |  | |  | | |
|  |  | | Mefloquin | | | | | |  | | |  | |  | | |
|  |  | | Others (…...……….....…) | | | | | |  | | |  | |  | | |
|  |  |  |  | | | | | |  | | |  | |  | | |
| E2 | No fever after treatment | | ………………………..…days | | | | | |  | | |  | |  | | |
| E3. | Parasite clearance after treatment | | ………………………..…days | | | | | |  | | |  | |  | | |
| E4 | Patient's condition at discharged | | No fever | | | | | | 1 | | |  | |  | | |
|  |  | | Stable | | | | | | 2 | | |  | |  | | |
|  |  | | Different (…...……….) | | | | | | 3 | | |  | |  | | |
| E5 | Discharge date (dd/mm/yy) | | …...../…...../...…… | | | | | |  | | |  | | | |  |
| **LABO** | | | | | | | | | | | | | | | |  |
| **No.** | **Contents** | | **Answers** | | | | | | **Code** | | | **Note** | | | |  |
| L1. | Real-time PCR results | | Negative | | | | | | 1 | | |  | | | |  |
|  |  | | Positive | | | | | | 2 | | |  | | | |  |
| L2. | Parasite load | | ……………………..copies/ml | | | | | |  | | |  | | | |  |
| L3. | Species | | *P.falciparum* | | | | | | 1 | | |  | | | |  |
|  |  | | *P.vivax* | | | | | | 2 | | |  | | | |  |
|  |  | | *P. malariae* | | | | | | 3 | | |  | | | |  |
|  |  | | *P. ovale* | | | | | | 4 | | |  | | | |  |
|  |  | | *P. knowlesi* | | | | | | 5 | | |  | | | |  |
| L4. | Drug resistant gene results | | Yes | | | | | | 1 | | |  | | | |  |
|  |  | | No | | | | | | 2 | | | - finish | | | |  |
| L5 | Types of drugs | | Artemisinin | | | | | | 1 | | |  | | | |  |
|  |  | | Lumefantrine | | | | | | 2 | | |  | | | |  |
|  |  | | Mefloquine | | | | | | 3 | | |  | | | |  |
|  |  | | Quinine | | | | | | 4 | | |  | | | |  |
|  |  | | Chloroquin | | | | | | 5 | | |  | | | |  |
|  |  | | Others (…………………….) | | | | | | 6 | | |  | | | |  |
| L6 | Gene encodes | | …………………….……… | | | | | |  | | |  | | | |  |
