# Supplementary material for: Understanding the epidemiology, clinical characteristics, knowledge and barriers to treatment and prevention of malaria among returning international laborers in northern Vietnam: a mixed-methods study
Source: BMC Infect Dis. 2022 May 13;22:460. doi: 10.1186/s12879-022-07322-5 (PMC9102356; doi:10.1186/s12879-022-07322-5)
Supplement: Supplementary file 3 — Additional file 3. Table S1: Primers and probes used for laboratory procedures. [file 12879_2022_7322_MOESM3_ESM.docx]

Table S1. Primers and probes for PCR

| ***P. knowlesi*** | NVPK-F | GCATCATAATCCAGTTTTATG |
| --- | --- | --- |
|  | NVPK-R | TACCTTGTACCTAATAATACTTGG |
|  | NVPK-P | **FAM**-CAGGGAATAGAGGGTTG-**MGB** |
| ***P. falciparum*** | Fal F | TATTGCTTTTGAGAGGTTTTGTTACTTTG |
|  | Fal R | ACCTCTGACATCTGAATACGAATGC |
|  | Fal P | **FAM**- ACGGGTAGTCATGATTGAGTT- **BHQ** |
| ***P. vivax*** | Viv_F | GCTTTGTAATTGGAATGATGGGAAT |
|  | Viv_R | ATGCGCACAAAGTCGATACGAAG |
|  | Viv_P | **HEX**- AGCAACGCTTCTAGCTTA- **BHQ** |
| ***P. ovale*** | Ova- F | CCG ACT AGG TTT TGG ATG AAA GAT TTT T |
|  | Ova- R | CAA CCC AAA GAC TTT GAT TTC TCA TAA |
|  | Ova- P | **VIC**-CGA AAG GAA TTT TCT TAT T- **MGB** |
| ***P. malariae*** | Mal-F | AGT TAA GGG AGT GAA GAC GAT CAG A |
|  | Mal-R | CAA CCC AAA GAC TTT GAT TTC TCA TAA |
|  | Mal-P | **FAM**-ATG AGT GTT TCT TTT AGA TAG C- **MGB** |
